# The Defects of Epigenetic Reprogramming in Dox-Dependent Porcine-iPSCs

**DOI:** 10.3390/ijms231911941

**Published:** 2022-10-08

**Authors:** Aiwen Jiang, Yangyang Ma, Xue Zhang, Qianqian Pan, Pengfei Luo, Hongyun Guo, Wangjun Wu, Juan Li, Tong Yu, Honglin Liu

**Affiliations:** 1 Department of Animal Genetics, Breeding and Reproduction, College of Animal Science and Technology, Nanjing Agricultural University, Nanjing 210095, China; 2 Anhui Province Key Laboratory of Local Livestock and Poultry, Genetical Resource Conservation and Breeding, College of Animal Science and Technology, Anhui Agricultural University, Hefei 230036, China

**Keywords:** porcine-iPSCs, epigenetic modification, ATAC-seq, DNA methylation, differential allelic expression gene

## Abstract

Porcine-induced pluripotent stem cells (piPSCs) are of great significance to animal breeding and human medicine; however, an important problem is that the maintenance of piPSCs mainly depends on exogenous expression of pluripotent transcription factors (TFs), and germline transmission-competent piPSCs have not yet been successfully established. In this study, we explore the defect of epigenetic reprogramming during piPSCs formation, including chromatin accessibility, DNA methylation, and imprinted gene expression, with high-throughput sequencing (ATAC-seq, WGBS, RNA-seq, and Re-seq) methods. We found the somatic features were successfully silenced by connecting closed chromatin loci with downregulated genes, while DNA methylation has limited effects on somatic silence. However, the incomplete chromatin remodeling and DNA demethylation in pluripotency genes hinder pluripotent activation, resulting in the low expression of endogenous pluripotency genes. In addition, the expression of potential imprinted genes was abnormal, and many allelic-biased expressed genes in porcine embryonic fibroblasts (PEFs) were erased, accompanied by establishment of new allelic-biased expressed genes in piPSCs. This study reveals the aberrant epigenetic reprogramming during dox-dependent piPSCs formation, which lays the foundation for research of porcine-iPSC reprogramming and genome imprinting.

## 1. Introduction

Waddington’s epigenetic landscape describes the formation of cell fate as a ball rolling down a hill, and it has different developmental choices at different times [1,2]. Therefore, embryonic stem cells (ESCs) derived from pre-implantation embryos can differentiate to all cell lineages during the developmental process [3,4]. In 2006, Yamanaka et al. found that terminally differentiated mouse embryonic fibroblasts (MEFs) could gain pluripotency after being reprogrammed by four transcription factors (TFs)-Oct4, Sox2, Klf4, and c-Myc (OSKM), and these cells were named to be induced pluripotent stem cells (iPSCs) [5]. The successful induction of iPSCs illustrates that the fate of cells can be reversed directly, and this process was divided into the initial, middle, and maturation phases [6,7,8]. Recently, many studies have explored the mechanisms involved in OSKM complete somatic reprogramming, and researchers have found epigenetic modification is involved in this process [9]. OSKM closes somatic loci and opens the pluripotent loci by interacting with chromatin [10], and stage-specific TFs play vital roles in this process [11]. Moreover, it is well documented that DNA methylation is a major barrier for mouse iPSC formation, and 5-azacytidine, the demethylation agent, can significantly promote iPSC generation [12].

Pigs are important economic animals, and porcine-iPSCs (piPSCs) are crucial for the development of agricultural production and human medicine [13,14]. A doxycycline (dox)-inducible system has been used to generate piPSCs; however, the low expression of endogenous pluripotent genes limits the application of piPSCs. In addition, the maintenance of piPSCs must rely on exogenous pluripotent TFs [15]. Recently, Gao et al. established porcine expanded potential stem cells (pEPSCs), which have good developmental potential in vitro [16]; however, germline chimeras from piPSCs or pEPSCs have not yet been produced. Considering the important role of epigenetic reprogramming in iPSC formation, studying the defect of epigenetic modification in dox-dependent piPSCs is necessary for producing exogenous TF-independent and germline transmission-competent piPSCs. 

Maternal and paternal genomes work unequally [17,18], and some genes are manifested through parent-of-origin-dependent differential expression or allelic-specific expression, known as “imprinted genes” [19,20]. For the past few years, next-generation RNA sequencing technology has been applied to identify imprinted genes [20,21,22,23]. At present, ~150 imprinted genes have been identified and validated in humans and mice successfully [24]. Normal genomic imprinting is necessary for the formation of primordial germ cells (PGCs) and early embryogenesis; genomic imprinting is relatively stable in somatic cells but sensitive to the reprogramming process, and it is frequently lost in human iPSCs [25,26,27]. The abnormal expression of imprinted genes may be why piPSCs cannot form germline chimeras. To date, imprinted genes in pigs are less researched, and how imprinted genes change after piPSC formation is unclear. 

In this study, we aimed to explore how chromatin accessibility and DNA methylation change after reprogramming, as well as how chromatin accessibility and DNA methylation regulate gene expression during piPSC formation through high-throughput sequencing (ATAC-seq, WGBS, and RNA-seq) approaches. In addition, we investigated parent-of-origin-dependent differentially expressed genes and parent-of-origin-dependent differentially open chromatin in PEF and piPSCs to research how the potential imprinted genes change after PEF reprogramming and whether allelic-specific open chromatin is related to the expression bias of imprinted genes. This study will provide a theoretical basis for the porcine reprogramming mechanism by connecting epigenetic modification with gene expression and will also supplement the information on porcine imprinted genes.

## 2. Results

### 2.1. Generation of piPSCs Genetically Matched with PEF

**Figure 1:**
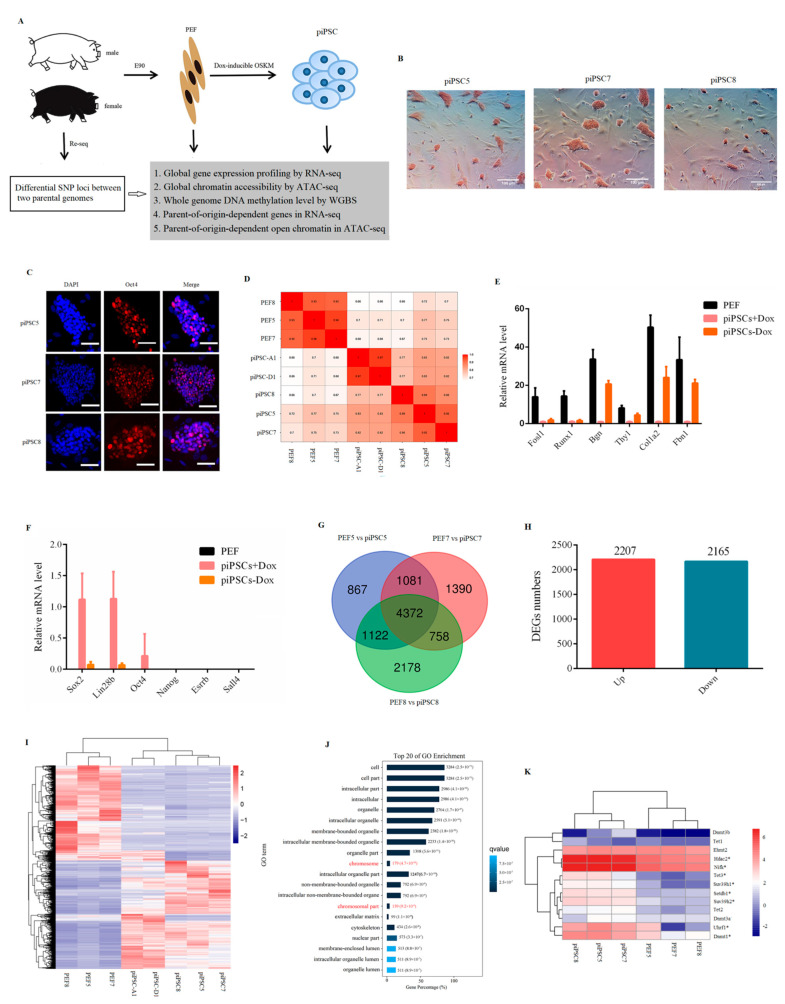
Generation of piPSCs genetically matched with PEFs. (**A**) Schematic for generation of genetically matched piPSCs from PEFs. (**B**) Alkaline phosphatase (AP) staining for three piPSCs. Scale bar, 100 µm. (**C**) Immunofluorescence analysis for Oct4 in three piPSCs. Nuclei were stained with DAPI. Scale bars, 100 µm. (**D**) Heatmap analysis for PEFs and piPSCs acquired in this study as well as piPSCs acquired in other groups based on global gene expression. (**E**) Relative mRNA level of PEF-specific genes. (**F**) Relative mRNA level of pluripotency genes. (**G**) Venn analysis of differential expressed genes between three groups. (**H**) Numbers of downregulated genes and upregulated genes between three groups. (**I**) Heatmap cluster for three PEFs and three piPSCs acquired in this study as well as two piPSCs acquired in other groups based on 4372 DEGs. (**J**) Top 20 of GO analysis terms for 4372 DEGs. (**K**) Heatmap analysis showing the expression level of epigenetic modification-related genes based on RNA-seq data. * *p* < 0.05 between PEFs and piPSCs.

Three PEF cell lines (PEF5, PEF7, PEF8) isolated from E90 porcine embryos were induced to pluripotency by transduction of doxycycline (dox)-dependent OSKM transcription factors (Figure 1A). In order to exclude the influence of sex on transcriptional variation, three PEF cell lines used in this study were male; when the packaging efficiency of FUW-mCherry was more than 90% (Figure S1A), and the infecting efficiency of FUW-mCherry was more than 80% (Figure S1B), the PEF cells infected with exogenous pluripotent factors were used for reprogramming. After seven to nine days of induction, colonies were isolated and expanded (Figure S1C). The result of alkaline phosphatase (AP) staining (Figure 1B) and immunofluorescence assay for Oct4 (Figure 1C) was positive. Following this, RNA-seq was applied to detect global gene expression between six cell lines in this study, as well as two piPSCs (piPSC-A1, piPSC-D1) reported in a previous study [28]. The results showed that all piPSCs were clustered together and were distinct from PEF cells (Figure 1D). The mRNA level of PEF-specific genes, e.g., *Fosl1*, *Runx1*, *Bgn*, *Thy1*, *Col1a2,* and *Fbn1*, was significantly downregulated after reprogramming. For endogenous pluripotency genes, *Sox2* and *Lin28b* were at relatively high level; however, the mRNA expression of *Oct4*, *Nanog*, *Sall4*, and *Esrrb* was at a low level after reprogramming (Figure 1E). After dox removal, the expression of PEF-specific genes was upregulated (Figure 1E) and all endogenous pluripotency genes were rapidly downregulated (Figure 1F). All results indicated that our three piPSCs were dox-dependent and they exhibited incomplete reprogramming features. 

Following this, to identify differentially expressed genes (DEGs) that could distinguish PEFs and piPSCs, we compared the transcriptional profiles of three piPSCs with their genetically matched PEF cells (PEF5 vs. piPSC5; PEF7 vs. piPSC7; PEF8 vs. piPSC8). The results showed that there were 3426 downregulated genes and 4016 upregulated genes in PEF5 vs. piPSC5 group (Table S1-1); for piPSC7, there were 3070 downregulated genes and 4531 upregulated genes compared with PEF7 (Table S1-2); for piPSC8, there were 4586 downregulated genes and 3844 upregulated genes compared with PEF8 (Table S1-3). There were 4372 genes which overlapped between three groups (Figure 1G and Table S1-4), including 2165 downregulated genes and 2207 upregulated genes (Figure 1H). Interestingly, all piPSCs could be totally separated from PEFs by these 4372 DEGs (Figure 1I). KEGG analysis of 4372 DEGs showed that “metabolic pathways,” “lysosome,” “cell cycle,” and “DNA replication” were significantly enriched in the top 20 (Figure S2), indicating the processes of cell metabolism and cell cycle were reprogrammed in three dox-inducible piPSCs. Interestingly, the GO analysis showed that chromosome-related terms were enriched in the top 20 of the GO analysis (Figure 1J). As shown in Figure 1K, genes associated with epigenetic modification, e.g., *Dnmt1*, *Suv39h2*, *Setdb1,* and *Hdac2*, were significantly changed after reprogramming, suggesting that during cell fate transition, epigenetic modifications (such as DNA methylation and chromatin structure) undergo drastic remodeling, which resulted in the changes in gene expression.

### 2.2. Accessible Chromatin Was Changed after Reprogramming

**Figure 2:**
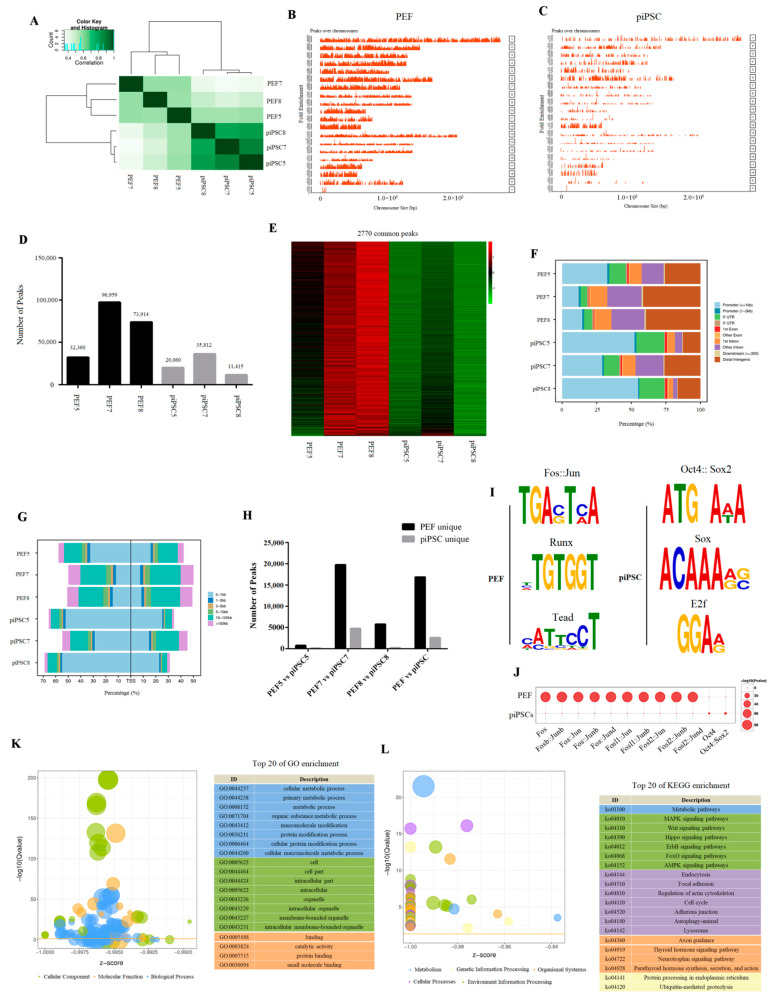
Differential chromatin accessibility between genetically matched PEFs and piPSCs. (**A**) Heatmap analysis of PEFs and piPSCs in ATAC-seq data. (**B**,**C**) Peaks over chromosomes in PEFs and piPSCs. (**D**) Number of peaks in each sample. (**E**) Heatmap analysis of 2770 common peaks in six samples. (**F**) Functional element analysis of accessible chromatin in PEFs and piPSCs. (**G**) Percentage of peaks near TSS in PEFs and piPSCs. (**H**) Numbers of PEF-unique peaks and piPSC-unique peaks in each group. (**I**) TF-motif analysis for PEF-unique peaks and piPSC-unique peaks. (**J**) Bubble diagram of differential TF-motif enrichment. (**K**) Top 20 terms of GO analysis for differential chromatin accessibility. (**L**) Top 20 terms of KEGG analysis for differential chromatin accessibility.

We then investigated accessible chromatin in PEFs and piPSCs by ATAC-seq. We obtained 71–95 million clean reads, and 28–45 million reads were uniquely mapped to the nuclear genome (*Sscrofa 11.1*) (Table S2). After peak calling, we found three piPSCs were clustered together and distinct from PEFs in ATAC-seq data (Figure 2A), indicating that the open chromatin region was changed after reprogramming. The peaks over the chromosome were significantly decreased in piPSCs compared to PEFs (Figure 2B,C), and we acquired 32,360; 96,959; 73,914; 20,060; 35,812; and 11,415 peaks in PEF5, PEF7, PEF8, piPSC5, piPSC7, and piPSC8, respectively (Figure 2D), which means the number of peaks were dramatically reduced in piPSCs compared to PEFs. A total of 2770 peaks collectively existed in six samples, and we found the chromatin accessibility for the most common peaks had declined after reprogramming (Figure 2E). All results illustrated that global chromatin is tighter after reprogramming. Although the number of peaks significantly decreased after reprogramming, the proportion of peaks located in the promoter and the percentage of peaks near transcription start sites (TSS, 0–1 kb) significantly increased in piPSCs compared to PEFs (Figure 2F,G), suggesting that the peaks open in piPSCs might play important roles in transcriptional regulation. 

We then explored the differential chromatin accessibility between PEFs and piPSCs. In the PEF5 vs. piPSC5 group, 4097 peaks were closed and 747 peaks were open after reprogramming; in the PEF7 vs. piPSC7 group, 19,756 peaks were closed and 4762 peaks were open after reprogramming; in the PEF8 vs. piPSC8 group, 5791 peaks were closed and 219 peaks were open after reprogramming (Figure 2H). We found there were large differences in the number of differential peaks between the three groups, which might be caused by individual differences and the different degree of reprogramming in the three piPSCs. In order to obtain as much open chromatin information as possible, we mixed ATAC-seq libraries of PEF5, PEF7, and PEF8 as the PEF library, and mixed ATAC-seq libraries of piPSC5, piPSC7, and piPSC8 as the piPSC library. Finally, we acquired 16,869 closed peaks and 2615 open peaks in the PEF vs. piPSC group (Figure 2H and Table S3). In general, the number of closed peaks was greater than the number of open peaks after reprogramming, which is consistent with the result that the total number of peaks in PEFs was much more than that in piPSCs (Figure 2D). A motif analysis for PEF-unique peaks showed that the accessible chromatin loci in PEF cells were mainly the DNA motifs for Fos/Jun, Runx, and Tead families, while piPSC-unique peaks were mainly the DNA motifs for the Oct4, Sox2, and E2f families (Figure 2I). Meanwhile, DNA motifs for the Fos/Jun family were significantly closed and DNA motifs for the Oct4/Sox2 family were significantly open after reprogramming (Figure 2J); this is consistent with the fact that somatic features were silenced and pluripotent features were activated during the reprogramming process. Functional analysis of differential peak-related genes showed a strong consistency with DEGs, and most differential peak-related genes were enriched in “cell metabolism,” “cell cycle,” and “lysosome” (Figure 2K,L), which further demonstrates the potential role of chromatin remodeling in regulating gene expression during dox-dependent piPSC formation.

### 2.3. Effect of Accessible Chromatin on Gene Expression

**Figure 3:**
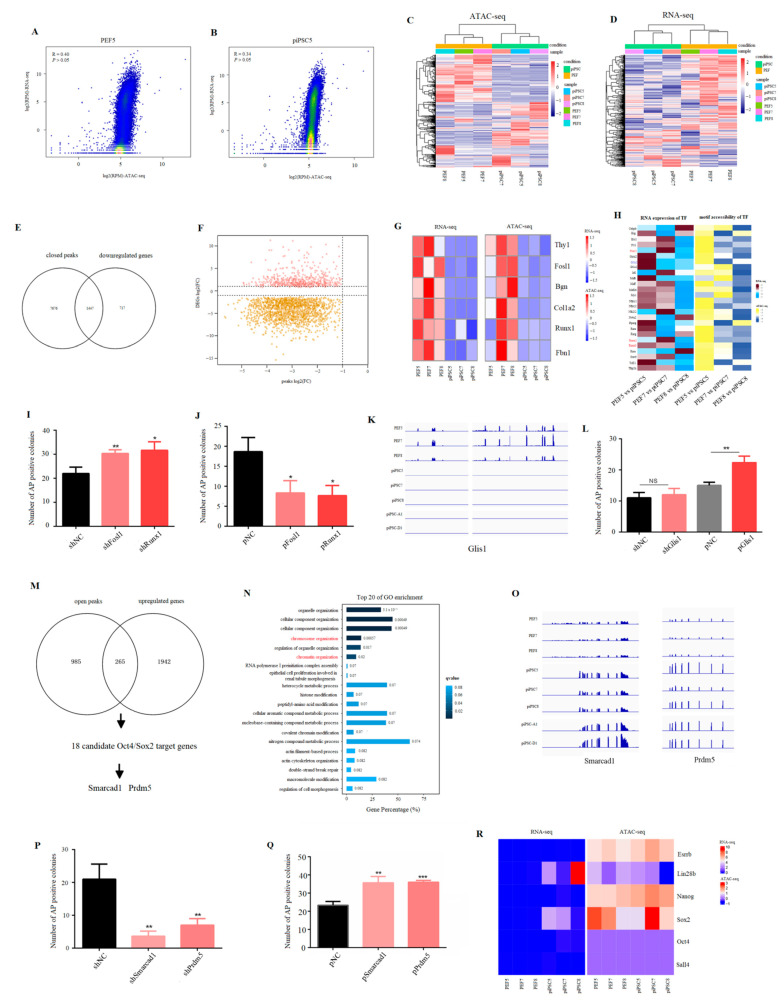
Effect of accessible chromatin on gene expression. (**A**,**B**) Scatter plots showing the correlation between ATAC-seq data and RNA-seq data in PEF5 and piPSC5. (**C**) Heatmap cluster of ATAC-seq data using DEGs. (**D**) Heatmap cluster of RNA-seq data using differentially open chromatin-related genes. (**E**) Venn diagram between downregulated genes and closed peak-related genes after reprogramming. (**F**) Scatter plot showing the correlation between closed peaks and DEGs. FC, fold change. (**G**) Heatmap analysis showing the changes of PEF marker genes—*Bgn*, *Runx1*, *Thy1*, *Fbn1* in ATAC-seq data and RNA-seq data. (**H**) Heatmap analysis of TFs whose expression was downregulated and motif accessibility was closed after reprogramming. (**I**) Number of AP positive colonies after Fosl1 shRNA and *Runx1* shRNA transfecting with OSKM. (**J**) Number of AP positive colonies after *Fosl1* and *Runx1* over-expression plasmid transfecting with OSKM. * *p* < 0.05, ** *p* < 0.01. (**K**) IGV analysis showing the expression level of *Glis1* after PEF reprogramming. (**L**) Number of AP positive colonies after *Glis1* shRNA and over-expression plasmid transfecting with OSKM. ** *p* < 0.01. (**M**) Venn diagram between upregulated genes and open peak-related genes after reprogramming. (**N**) Top 20 terms of the GO analysis for common genes between upregulated genes and open peak-related genes. (**O**) IGV graph showing the mRNA level of *Smarcad1* and *Prdm5* in RNA-seq data. (**P**) Number of AP positive colonies after *Smarcad1* shRNA and *Prdm5* shRNA transfecting with OSKM. ** *p* < 0.01 (**Q**) Number of AP positive colonies after *Smarcad1* shRNA and *Prdm5* over-expression plasmid transfecting with OSKM. ** *p* < 0.01, *** *p* < 0.001. (**R**) Heatmap analysis showing the changes in pluripotency genes—*Oct4*, *Sox2*, *Sall4*, *Nanog*, *Lin28b*, and *Esrrb* in ATAC-seq data and RNA-seq data.

To explore the correlation between global chromatin accessibility and global gene expression, we used a 100-kb window to scan the genome and reads mapped in ATAC-seq and RNA-seq were normalized by read per million (RPM). The results showed that there was low correlation between global chromatin accessibility and global gene expression in PEF cells and in piPSCs (Figure 3A,B and Figure S3). We then co-analyzed 4372 DEGs (Figure 1E) with differential peak-related genes (Figure 2H) to explore whether the change in accessible chromatin is involved in transcriptional regulation during reprogramming. The results showed that 4372 DEGs could separate three PEF cell lines from three piPSCs in ATAC-seq data (Figure 3C). Consistent with this result, PEFs and piPSCs could be correctly clustered in RNA-seq data using differentially open chromatin loci (Figure 3D). These results illustrated that the changes in chromatin accessibility were involved in the regulation of gene expression. After co-analyzing 2165 downregulated genes (Table S1-4) with closed peak-related genes (Table S3-1), we found 1447 downregulated genes (~66.74%) were associated with closed chromatin (Figure 3E), and the majority of closed peaks were involved in the downregulation of gene expression (Figure 3F). Furthermore, marker genes from PEF cell lines, such as *Bgn*, *Runx1*, *Thy1*, and *Fbn1* were identified in this process (Figure 3G), indicating exogenous OSKM silenced somatic features by closing somatic-specific chromatin.

In addition, we focused on the function of TFs whose gene expression and motif accessibility were both downregulated after reprogramming; the result showed that *Runx* and *Fosl* families were enriched in this process (Figure 3H), and the number of AP positive colonies was significantly upregulated after *Fosl1* or *Runx1* shRNA was co-transfected with exogenous OSKM (Figure 3I). Consistently, the number of AP positive colonies significantly declined after *Fosl1* or *Runx1* over-expression plasmid was co-transfected with exogenous OSKM (Figure 3J). Unexpectedly, *Glis1*, a positive factor in mouse iPSC formation [29], was also identified in this process (Figure 3H), and its expression was downregulated not only in our iPSCs (piPSC5, piPSC7, and piPSC8), but also in other iPSCs (piPSC-A1 and piPSC-D1) (Figure 3K). To understand the role of *Glis1* in piPSC formation, we detected the number of AP positive colonies after *Glis1* knock-down or over-expression. The result showed that the number of AP positive colonies was significantly upregulated after *Glis1* over-expression even though knock-down of *Glis1* had no effect (Figure 3L), suggesting the abnormal decrease in *Glis1* may be an obstacle for piPSC formation.

We then analyzed the relationship between upregulated genes (Table S1-4) and open peak-related genes (Table S3-2). Different from the process of somatic silencing, only 265 genes overlapped (Figure 3M). The GO analysis for 265 common genes found that “chromatin organization” and “chromosome organization” were significantly enriched in the top 20 (Figure 3N). Co-analysis of these 265 genes with Oct4/Sox2 tentative target genes (TTGs) published in a previous study [30] showed that 18 Oct4/Sox2 TTGs had open peaks and upregulated expression after reprogramming (Table S4). Of these, *Prdm5* and *Smarcad1* were identified as important epigenetic regulators (Figure 3M), and their expression was upregulated not only in our iPSC cells (piPSC5, piPSC7, and piPSC8), but also in other iPSCs (piPSC-A1 and piPSC-D1) (Figure 3O). The knocking down of *Prdm5* and *Smarcad1* significantly decreased the number of AP positive colonies, while over-expression of *Prdm5* and *Smarcad1* significantly increased the number of AP positive colonies (Figure 3P,Q). Even though the motif for Oct4/Sox2 was significantly open (Figure 2J), and partial pluripotency genes, e.g., *Sox2* and *Lin28b* were upregulated after reprogramming, their chromatin was not open (Figure 3R). All results indicated that incomplete chromatin remodeling for pluripotency genes may be the reason why expression of endogenous pluripotent genes is not activated.

### 2.4. Loss of Global DNA Methylation after piPSC Reprogramming

**Figure 4:**
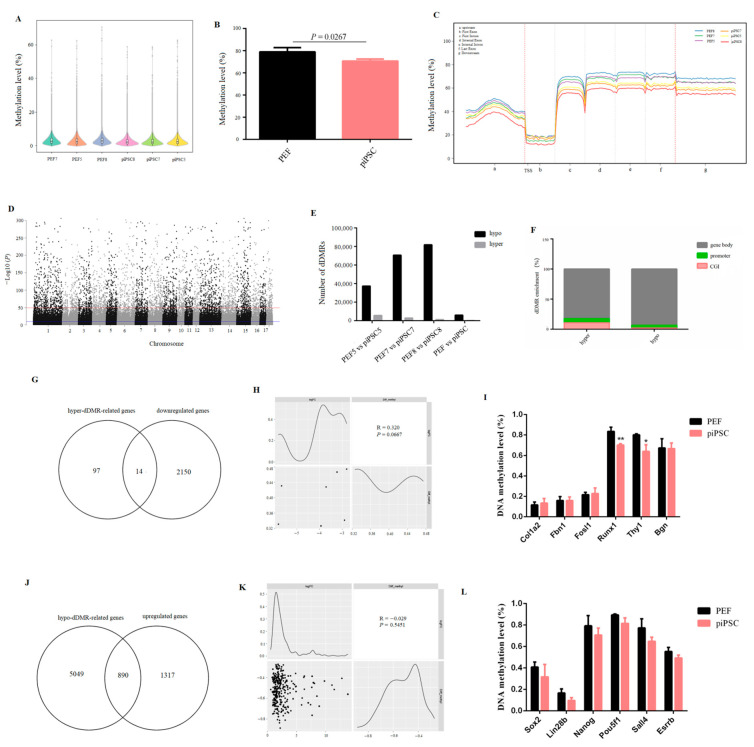
The global DNA methylation level was reduced after reprogramming. (**A**,**B**) The changes in genome-wide DNA methylation level after reprogramming. (**C**) Map of DNA methylation level in transcription elements. (**D**) Manhattan plot of the genome-wide *P*-values for the association between the methylation level and reprogramming. (**E**) Numbers of dDMRs in each group. (**F**) The distribution of dDMRs in functional elements. (**G**) Venn diagram between downregulated genes and hyper-dDMR-related genes after reprogramming. (**H**) Correlation analysis showing the relationship between downregulated genes and hyper-dDMRs in promoter. (**I**) The changes in DNA methylation level in PEF-specific genes after reprogramming. * *p* < 0.05, ** *p* < 0.01. (**J**) Venn diagram between upregulated genes and hypo-dDMR-related genes after reprogramming. (**K**) Correlation analysis showing the relationship between upregulated genes and hypo-dDMRs in promoter. (**L**) The changes of DNA methylation level in pluripotency genes after reprogramming.

DNA methylation is an important epigenetic modification in regulating the binding activity of TFs and its gene expression [31]. To investigate the role of DNA methylation in PEF reprogramming, a genome-wide DNA methylation level was detected by whole genome bisulfite sequencing (WGBS) for six samples, and high-quality sequencing data were obtained (Table S5). The result showed that the whole genome DNA methylation level was significantly decreased after reprogramming (Figure 4A,B). In general, the DNA methylation level was dramatically reduced at TSS, and then maintained at a stably high level in gene bodies (Figure 4C), and this conforms to the general characteristics of gene transcriptional expression. We then identified differentially DNA-methylated regions (dDMRs), and 70,693 dDMRs were obtained (Figure 4D). Most regions were hypomethylated with a few regions that were hypermethylated in three groups (Figure 4E, Tables S6–S8), which is in accordance with the loss of global DNA methylation after reprogramming (Figure 4A). The functional element analysis showed that the distribution of dDMRs was mainly located in the gene body (~80%), whether in hypermethylated regions (hyper-dDMRs) or in hypomethylated regions (hypo-dDMRs). Meanwhile, the ratio of hyper-dDMRs distributed in promoter and CG islands (CGI) was higher than that of hypo-dDMRs (Figure 4F), and this is consistent with the DNA-methylation dynamics of embryonic skeletal muscle [32], indicating that this could be a general feature of mammals’ DNA methylation pattern.

We then performed a combined analysis of dDMR-associated genes with DEGs to explore the role of DNA methylation in PEF reprogramming. For the process of somatic silencing, we obtained 111 hyper-dDMR-related genes which commonly existed in three groups (Table S9-1); however, only 14 genes overlapped between hype-dDMR-related genes and downregulated genes (Figure 4G). In addition, only seven downregulated genes were accompanied by promoter hypermethylation; there was no significant correlation between promoter hypermethylation and gene down-expression (Figure 4H). The promoter methylation level of PEF-specific genes, e.g., *Bgn1*, *Fbn1*, *Runx1*, *Fosl1*, *Thy1*, and *Col1a2*, whose expression was downregulated in our dox-iPSCs, was not upregulated after reprogramming (Figure 4I and Figure S4). All results suggested that the changes in DNA methylation play limited roles in downregulating PEF-specific genes. 

In addition, we obtained 5939 hypo-dDMR-related genes which commonly existed in three groups (Table S9-2), and we found 890 hypomethylated genes (~40%) overlapped with upregulated genes (Figure 4J). The upregulation of most genes was accompanied by gene hypomethylation, even though there was no significant correlation between promoter hypomethylation and genes upregulation (Figure 4K). The methylation level of all pluripotency genes was not changed (Figure 4L). Interestingly, we found the promoter region of *Sox2* and *Lin28b* exhibited less than 5 mC distribution, and their expression was significantly upregulated in our dox-dependent iPSCs. Other pluripotency genes, e.g., *Oct4*, *Nanog*, *Esrrb,* and *Sall4*, showed a high density of 5 mC distribution and their expression failed to upregulate. (Figure 4L and Figure S5). These results illustrated that DNA methylation is a major obstruction for the activation of pluripotency genes, and that incomplete DNA demethylation impedes the acquisition of pluripotency.

### 2.5. Identification of Allele-Biased Expressed Genes and Allele-Biased Open Chromatin

**Figure 5:**
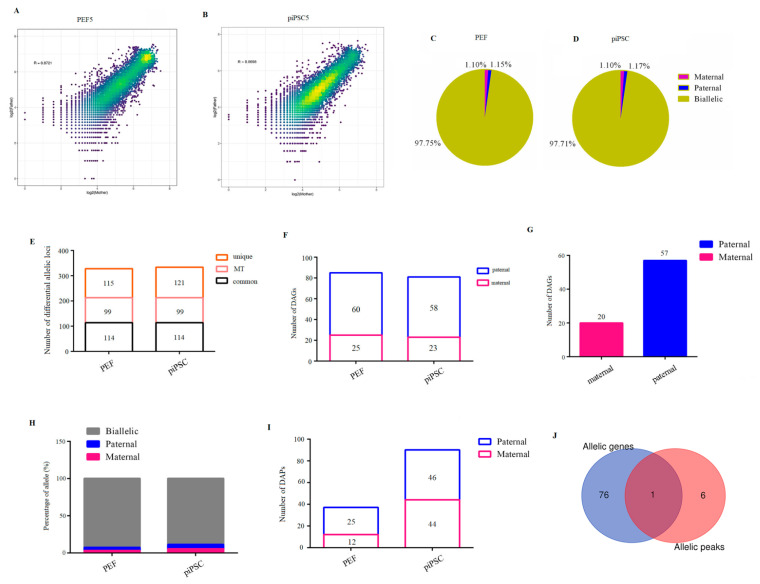
Identification of parent-of-origin-dependent genes and parent-of-origin-dependent accessible chromatin. (**A**,**B**) Scatter plots show comparable transcription activities between two parental genomes in PEF5 (**A**) and piPSC5 (**B**). (**C**,**D**) Percentage of maternal-expression and paternal-expression loci in three PEF cell lines (**C**) and three piPSCs (**D**) for RNA-seq data. (**E**) Numbers of differential allelic loci in PEFs and piPSCs for RNA-seq data. (**F**) Numbers of paternal differential allelic genes in PEF-unique loci and piPSC-unique loci, respectively. (**G**) Numbers of differential allelic genes in PEF- and piPSC-common loci. (**H**) Percentage of maternal- and paternal-allele open loci in three PEF cell lines and three piPSCs for ATAC-seq data. (**I**) Number of differential allelic peaks in PEFs and piPSCs, respectively. (**J**) Overlap of parent-of-origin-dependent genes and parent-of origin-dependent peak-related genes.

Finally, we blasted parental discrepant polymorphic SNP loci into RNA-seq data, and we acquired 18,916 SNP loci whose Chi-square test significantly deviates from 1:1 (Table S10). The two parental genomes showed comparable transcription activities in PEFs or piPSCs (Figure 5A,B and Figure S6). To acquire credible allele-biased expressed genes, allelic loci with a threefold difference between male and female parents were screened. As a result, we identified 1.10% maternal allele-biased expression loci and 1.15% paternal allele-biased expression loci in the PEF group (Figure 5C). Consistently, 1.10% and 1.17% loci were found to be maternal allele-biased expression and paternal allele-biased expression in the piPSCs group, respectively (Figure 5D). Expectedly, all 98 mitochondrial loci are from the maternal alleles (Figure 5E and Table S11), demonstrating the reliability of our analysis. There were 114 differential allelic loci which commonly existed in PEFs and piPSCs; moreover, 115 differential allelic loci were specific to PEFs and 121 differential allelic loci were specific to piPSCs (Figure 5E). There were 25 maternally expressed genes and 60 paternally expressed genes for 115 PEF-unique loci (Figure 5F and Table S12). There were 23 maternally expressed genes and 58 paternally expressed genes for 121 piPSC-unique loci (Figure 5F and Table S13). These results suggested potential imprinted genes were unstable and susceptible after piPSC formation. For 114 common loci in PEFs and piPSCs, we found 20 maternally expressed genes and 57 paternally expressed genes (Figure 5G and Table S14), and these genes have important potential imprinted genes for porcine development.

To understand whether the allelic imbalance of allele-biased expressed genes is regulated by allele-biased open chromatin, we researched parent-of-origin-dependent accessible chromatin after blasting parental discrepant SNP loci into ATAC-seq data. We acquired 3223 SNP loci in ATAC-seq data whose parental chromatin loci could be totally separated in ATAC-seq data (Table S15). We found 3.29% maternal allele-biased open loci and 4.22% paternal allele-biased open loci in PEFs, accounting for 12 maternally open peaks and 25 paternally open peaks in PEFs (Figure 5H,I and Table S16). A total of 5.24% maternal allele-biased open loci and 5.71% paternal allele-biased open loci were found in piPSCs, accounting for 44 maternally open peaks and 46 paternally open peaks in piPSCs (Figure 5H,I and Table S17). After co-analysis of differential allelic genes and differential allelic peaks, we found only one gene was overlapped between allele-specific ATAC-seq signals and allele-specific genes (Figure 5J), indicating the bias of allelic open chromatin played a limited role in expression bias of imprinted genes. 

## 3. Discussion

To investigate the underlying mechanism of pluripotent genes’ low expression in dox-dependent piPSCs, we analyzed the relationship between epigenetic modifications and gene expression in PEFs and piPSCs. Studies reported that the reprogramming method and sex are associated with transcriptional patterns of iPSCs [33,34,35]. In this study, to ensure that the difference between PEF and piPSCs was due to the process of reprogramming,, we gained three piPSCs (piPSC5, piPSC7, piPSC8) with the same sex and same reprogramming method. We found good repeatability among three PEF cell lines or piPSCs in RNA-seq data, and DEGs obtained in this study separated PEF cells not only from genetically matched piPSCs (piPSC5, piPSC7, piPSC8), but also from other piPSCs (piPSC-A1 and piPSC-D1). The process of somatic reprogramming is a comprehensive result involved in metabolic reprogramming, cell cycle reprogramming, and epigenetic reprogramming [36]. We found that 4372 DEGs were significantly enriched in cell cycle, cellular metabolism, and chromatin structure pathways. It is well documented that epigenetic modification regulates gene expression by changing chromatin dynamics [37], and we found epigenetic regulators, such as *Dnmt1*, *Suv39h1*, and *Hdac2* were mapped in DEGs. This makes us believe that the transcriptional difference between PEFs and piPSCs may be regulated by epigenetic modification, and studying the changes of epigenetic modification during piPSC formation is important to solve the difficulties of piPSCs.

ATAC-seq data revealed that a large number of peaks were closed, accompanied by a small number of peaks that were open after reprogramming. This illustrated that global chromatin accessibility decreased and chromatin structure was tighter in piPSCs than in PEFs, which is in accordance with miPSC [11]. The whole genome DNA methylation level was significantly decreased after reprogramming. For the past few years, researchers have studied the relationship between chromatin remodeling and gene expression in iPSCs and ESCs, and they found chromatin remodeling determines cell fate and colony formation [10,38,39]; however, Veazey et al. found that changes in the chromatin structure do not always influence the expression of candidate genes [40]. In this study, we found there was a low association between global transcriptional expression and global accessible chromatin. Furthermore, some closed loci showed increased gene expression, which further confirmed the chromatin structure was not always consistent with transcriptional expression. The way in which gene expression is upregulated when chromatin is turned off needs further study. 

Li et al. found during MEF reprogramming that chromatin loci enriched with motifs for MEF-specific TFs are quickly closed, followed by a slower opening up of pluripotent chromatin loci [10], and DNA methylation is also involved in this process [12]. In this study, we found downregulation of PEF-specific genes was initiated by the close of peaks, and this process is similar to MEF reprogramming [10]. The promoter’s DNA methylation level of PEF-specific genes was not upregulated, although their expression and chromatin accessibility were significantly downregulated. These results suggest that the silence of somatic features was mainly regulated by chromatin remodeling, not DNA methylation. In addition, the chromatin of pluripotent genes was not open; the promoter’s DNA methylation level of *Oct4* and *Nanog*, whose expression was regulated by DNA methylation [36], was not reduced, suggesting incomplete chromatin remodeling and DNA demethylation results in the low expression of endogenous pluripotent gene. Motif enrichment of Oct4/Sox2 families was increased in piPSCs, and this is consistent with the fact that they were transfected into PEFs. These results suggest the opening of the Oct4/Sox2 motif relies on exogenous OSKM. 

Genome imprinting is necessary for the development and reproduction of mammals [41], and studies have found it is controlled by differential DNA methylation [42]. In mouse early embryos, maternal and paternal genomes showed largely comparable allelic gene expression and allelic open chromatin landscapes; a similar imprinting pattern exists between iPSCs and their somatic cell [34]. In this study, chromatin accessibility and allelic gene expression between two parental genomes exhibited a very small difference, and parent-of-origin-dependent differentially accessible chromatin played a limited role in the expression bias of allelic genes, which is consistent with previous studies [43]. Even so, we found a list of allelic-biased expressed genes which commonly existed in PEFs and piPSCs, and these genes may be important for porcine development; however, further studies are needed to verify the conservation of these genes in other animals, such as humans and mice. In addition, the expression of potential imprinted genes is abnormal, and many allelic-biased expressed genes in PEF cells are erased, accompanied by the establishment of new allelic-biased expressed genes in piPSCs. The correct erasure and establishment of imprinted genes are essential for embryo development, and improper expression of imprinted genes might be a potential reason for the unsuccessful establishment of piPSCs-origin germline chimera.

In summary, our study found global chromatin accessibility was closed, whole genome DNA methylation was reduced, and the expression of parental differential allelic genes was abnormal after reprogramming. Exogenous OSKM closed somatic specific chromatin loci and downregulated PEF specific genes. DNA methylation plays a limited role in silencing somatic features; however, exogenous OSKM could not open pluripotent chromatin loci, and promoter demethylation was incomplete in pluripotent genes. Incomplete chromatin remodeling and DNA demethylation result in the low expression of endogenic pluripotent genes, and the opening of the Oct4/Sox2 binding motif must depend on an exogenous OSKM. More importantly, we obtained a list of differential allelic genes which commonly existed in PEFs and piPSCs, and confirmed that the expression of differential allelic genes is not controlled by parental differential open chromatin. This study provides a theoretical basis for the porcine reprogramming mechanism, and also improves the information on porcine genomic imprinting, which is of great significance in the genetic breeding of mammals.

## 4. Materials and Methods

### 4.1. Animals 

*Large White* and *Meishan* pigs served as male and female parents, respectively; at 90 days after mating, the *Meishan* sow was slaughtered to obtain PEFs. C57BL/6J mice were slaughtered at 12.5 days after pregnancy to obtain MEFs. All procedures were performed according to the Institutional Animal Care and Use Committee of Nanjing Agricultural University, Nanjing, China (SYXK2011-0036).

### 4.2. Cell Culture 

To obtain PEFs, the tip of the ears from 90 day-old porcine embryos (E90) were digested by trypsin for 30 min at 37 °C. MEFs were isolated from 13.5 days from mouse embryos (E13.5). The cultural method used to obtain PEFs and MEFs can be found in our previous study [28]. 

### 4.3. Generation of piPSCs

Three PEFs (PEF5, PEF7, PEF8) within 3 passages were plated in 6-well plates before iPSC induction. The 293T cells were plated on a 10cm dish with 2 × 10^7^ cells. After adherence, the lentiviral vector of FUW-mCherry (#138531, AddGene, Cambridge, MA, USA), TetO-FUW-OSKM (#20321, AddGene,), and FUW-M2rtTA (#20342, AddGene,) were transfected into 293T cells, respectively, by Lipofectamine^®^ 3000 Transfection Reagent (L3000015, Invitrogen™, Carlsbad, CA, USA). At 48 h after transfection, the lentiviral particles were collected and mixed with polybrene (8 µg/mL, 107689-10G, Sigma Aldrich, St. Louis, MO, USA). PEF was infected by lentiviral particles containing FUW-mCherry, or an equal mixture of TetO-FUW-OSKM and FUW-M2rtTA, for 24 h. When the infectivity of FUW-mCherry reached 80%, the cells infected with the mixture of TetO-FUW-OSKM and FUW-M2rtTA were re-plated on a 60 mm dish and the cells were cultured in LF2i medium for 7–9 days to acquire colonies. The colonies were picked up and seeded on 12-well plates coated with MEF feeders. The packaging efficiency and infecting efficiency rates were calculated by the fluorescence expression level of FUW-mCherry, and they are determined as the ratio of the number of FUW-mCherry positive cells to total cells.

### 4.4. Alkaline Phosphatase (AP) Staining

Stem cells show high alkaline phosphatase (AP) activity, which decreased after differentiation. In this study, AP staining was used to measure AP activity. Briefly, AST Fast Red TR (F6760, Sigma Aldrich) and α-Naphthol AS-MX Phosphate (N4875-1G, Sigma Aldrich) were used to detect AP activity. Ice-cold PBS (SH30256.01B, Hyclone Laboratories) was used to wash cells twice; after being fixed with 4% paraformaldehyde, the cells were then incubated with the mixture of Fast Red TR and Naphthol AS-MX for 10 min. 

### 4.5. Immunofluorescence Assay 

To perform the immunofluorescence analysis, 4% paraformaldehyde was used to fix the cells. The fixed cells were then immunostained with rabbit antibody against Oct4 (SC-5279, Santa Cruz Biotechnology, Santa Cruz, CA, USA) after being permeabilized and blocked. After washing with PBS three times, cells were incubated with a Rhodamine (TRITC)-conjugated goat anti-rabbit IgG (1:100 dilution, ZSGB-BIO) for 1 h. After being stained with 4, 6-diamidino-2-phenylindole-dihydro-chloride (DAPI) for 15 min, the images of cells were captured using a Zeiss LSM 710 META confocal microscope.

### 4.6. Library Construction and Sequencing

In this study, 8 RNA-seq libraries (PEF5, PEF7, PEF8, piPSC5, piPSC7, piPSC8, piPSC-A1, and piPSC-D1), 6 ATAC-seq and WGBS libraries (PEF5, PEF7, PEF8, piPSC5, piPSC7, and piPSC8) and 2 re-sequencing (Re-seq) libraries (father, mother) were constructed, sequenced, and analyzed by Annoroad (Beijing, China), and the information regarding the construction of the libraries can be found in a previous study [32,44,45].

### 4.7. Identification of Parent-of-Origin-Dependent Genes

To identify parent-of-origin-dependent genes, the different SNP loci in the 2 parents chromosome were filtrated by deleting the loci where one SNP corresponds to multiple sequences. The RNA-seq data were then filtered by deleting the inconsistent loci in 3 replicates. Loci with more than 10 allelic reads in each sample were then reserved. Finally, the loci which significantly deviated from 1:1 performed by Chi-square test were used for analysis. The allele-specific genes were identified by at least threefold change between the numbers of maternal and paternal reads. 

### 4.8. Identification of Parent-of-Origin-Dependent Accessible Chromatin Regions

The identification of the allele-specific accessible chromatin regions was consistent with the identification of imprinted genes in transcriptome. The SNP sites screened by Re-seq data were intersected with the original ATAC-seq data. According to these SNP sites, the origin of genotypes can then be determined according to the similarities and differences between the genotypes of PEF5, PEF7, PEF8, piPSC5, piPSC7, and piPSC8 and the parent genotypes.

### 4.9. RNA Oligonucleotides, Cell Transfection, and Real-Time Quantitative PCR

The shRNA and over-expression plasmids were purchased from GenePharma (Shanghai, China). Their sequences were listed in Table S18. The methods of RNA extraction and qPCR can be found in a previous study [46].The primer sequences of the primers are listed in Table S19.

### 4.10. Statistical Analysis

Statistical analyses were performed using Prism 6 software (GraphPad Software, La Jolla, CA, USA). Results were expressed as means ± SD, and error bars represent the SD of 3 replicates, unless stated otherwise. Data were compared using the 2-tailed unpaired Student’s *t* test. The value of *p* < 0.05 was considered significant.

## Data Availability

Sequencing data for PEF5, piPSC5, PEF7, piPSC7, PEF8, and piPSC8 have been submitted to the NCBI Gene Expression Omnibus (GEO) with accessions: GSE173275 for ATAC-seq data, GSE173304 for RNA-seq data; parental genome re-seq data have been submitted to Sequence Read Archive (SRA) with BioProject number: PRJNA727677; RNA-seq data for piPSC-A1 and D1 were submitted to SRA with BioProject number: PRJNA737304. Sequencing data for DNA methylation are available from authors.

## Supplementary Materials

The following supporting information can be downloaded at: https://www.mdpi.com/article/10.3390/ijms231911941/s1.
