# Supplementary material for: The Defects of Epigenetic Reprogramming in Dox-Dependent Porcine-iPSCs
Source: Int J Mol Sci. 2022 Oct 8;23(19):11941. doi: 10.3390/ijms231911941 (PMC9570186; doi:10.3390/ijms231911941)
Supplement: Supplementary file 1 [file ijms-23-11941-s001.zip › supplemental figures.pdf]

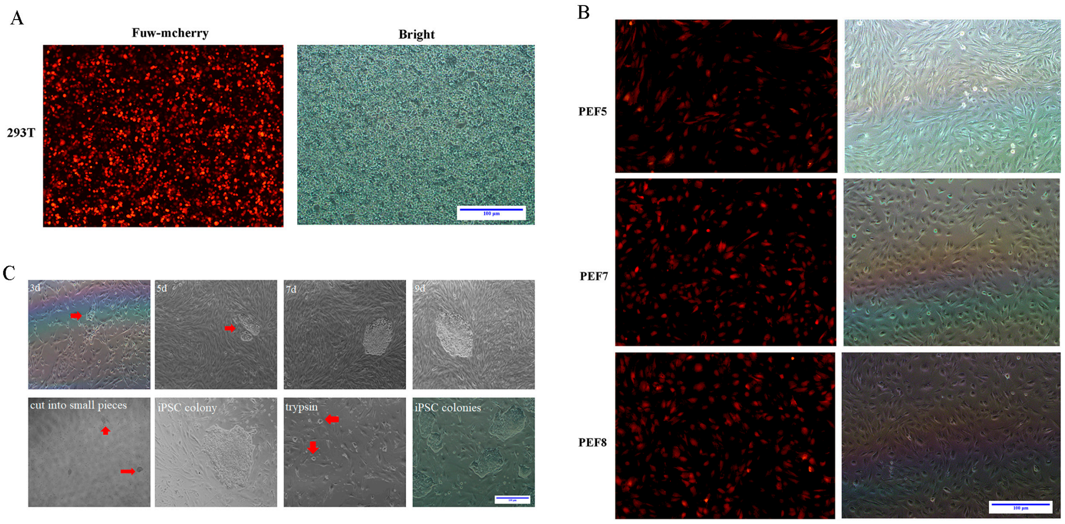

Figure S1 Efficiency of packaging and infecting virus of Fuw-mcherry. (**A**) Fuw-mcherry plasmid was packaged into lentivirus plasmid by 293T cells. Successful package of Fuw-mcherry showed red, and cell showed in Bright. Scar Bar, 100 μm. (**B**) Virus carrying Fuw-mcherry was used to infected PEF5, PEF7 and PEF8; successful infection of cells showed red, and cell showed in Bright. Scar Bar, 100μm. (**C**) The process of piPSC formation.

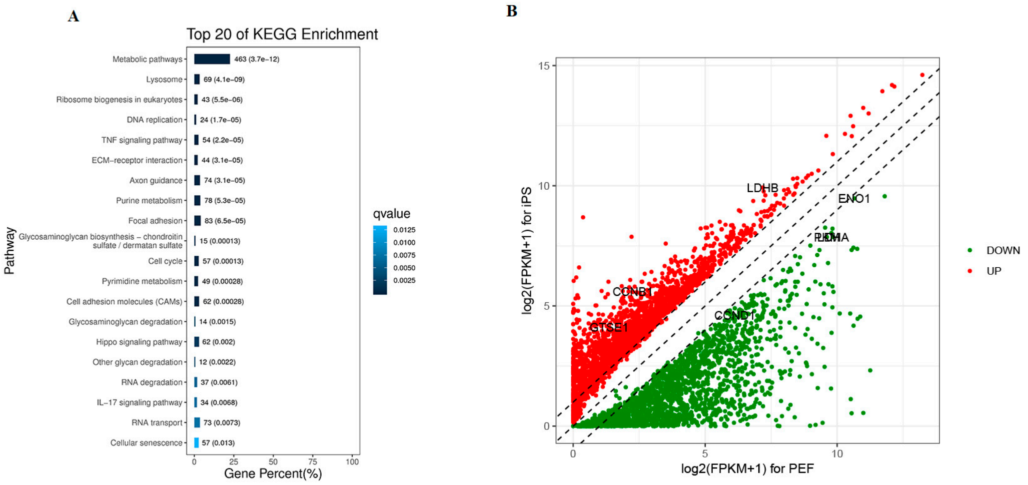

Figure S2 KEGG analysis for 4,372 DEGs. (**A**) Top 20 terms of KEGG analysis for 4,372 DEGs. (**B**) Scatter plot showing the expression of genes enriched in “metabolic pathway” “cell cycle” and “DNA replication” by KEGG analysis.

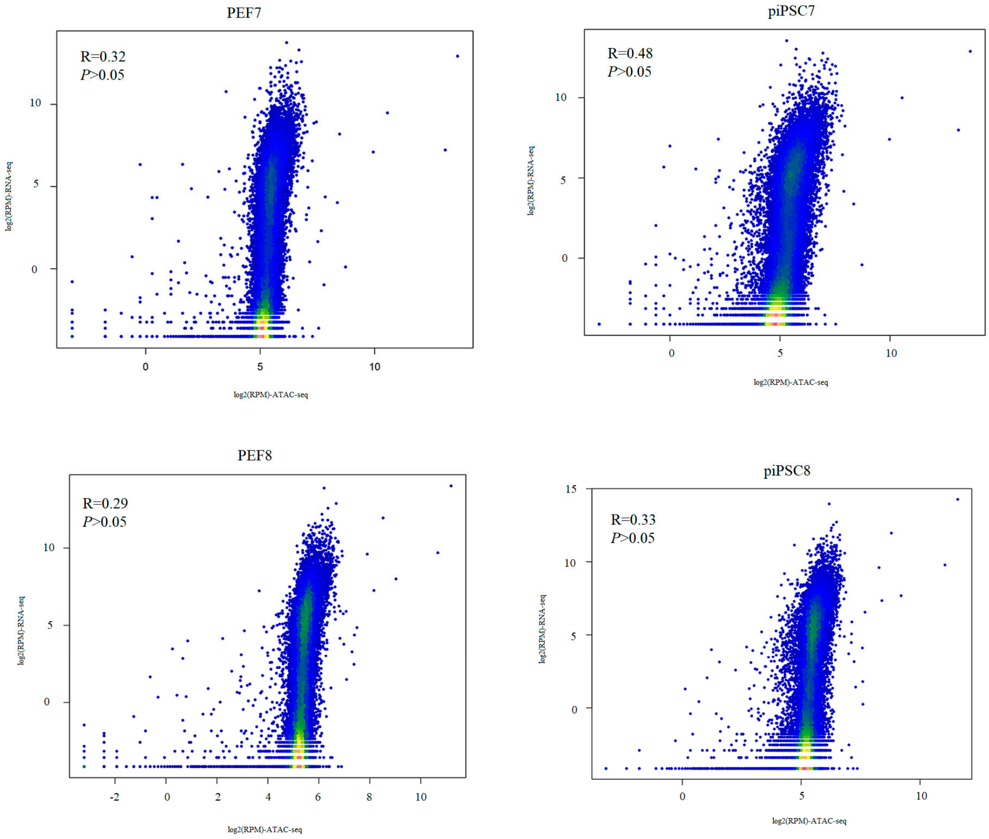

Figure S3 Scatter plots showing the correlation between ATAC-seq data and RNA-seq data. (**A**) Scatter plots of PEF7 between ATAC-seq data and RNA-seq data. (**B**) Scatter plots of PEF8 between ATAC-seq data and RNA-seq data. (**C**) Scatter plots of piPSC7 between ATAC-seq data and RNA-seq data. (**D**) Scatter plots of piPSC8 between ATAC-seq data and RNA-seq data.

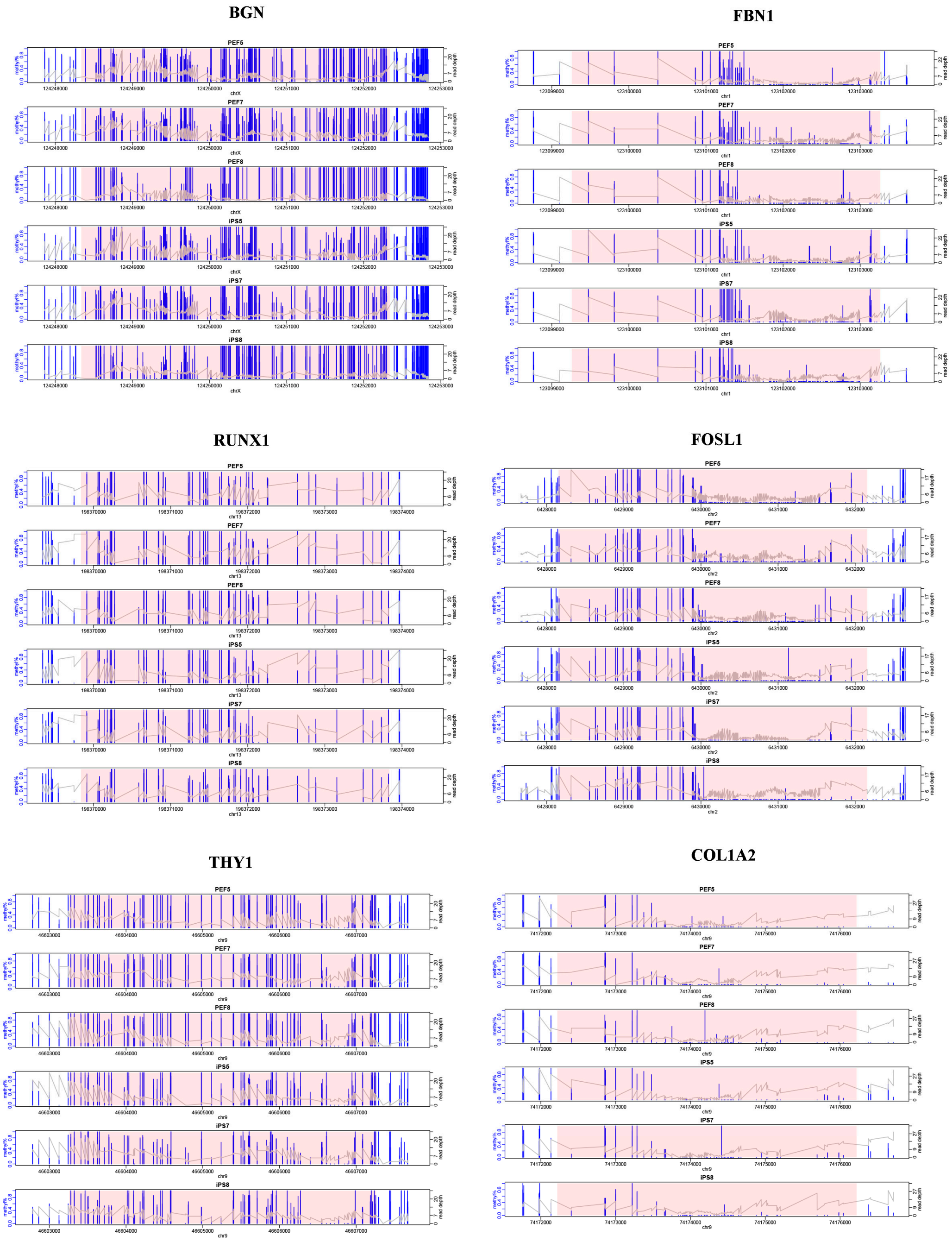

Figure S4 DNA methylation level of PEF specific genes

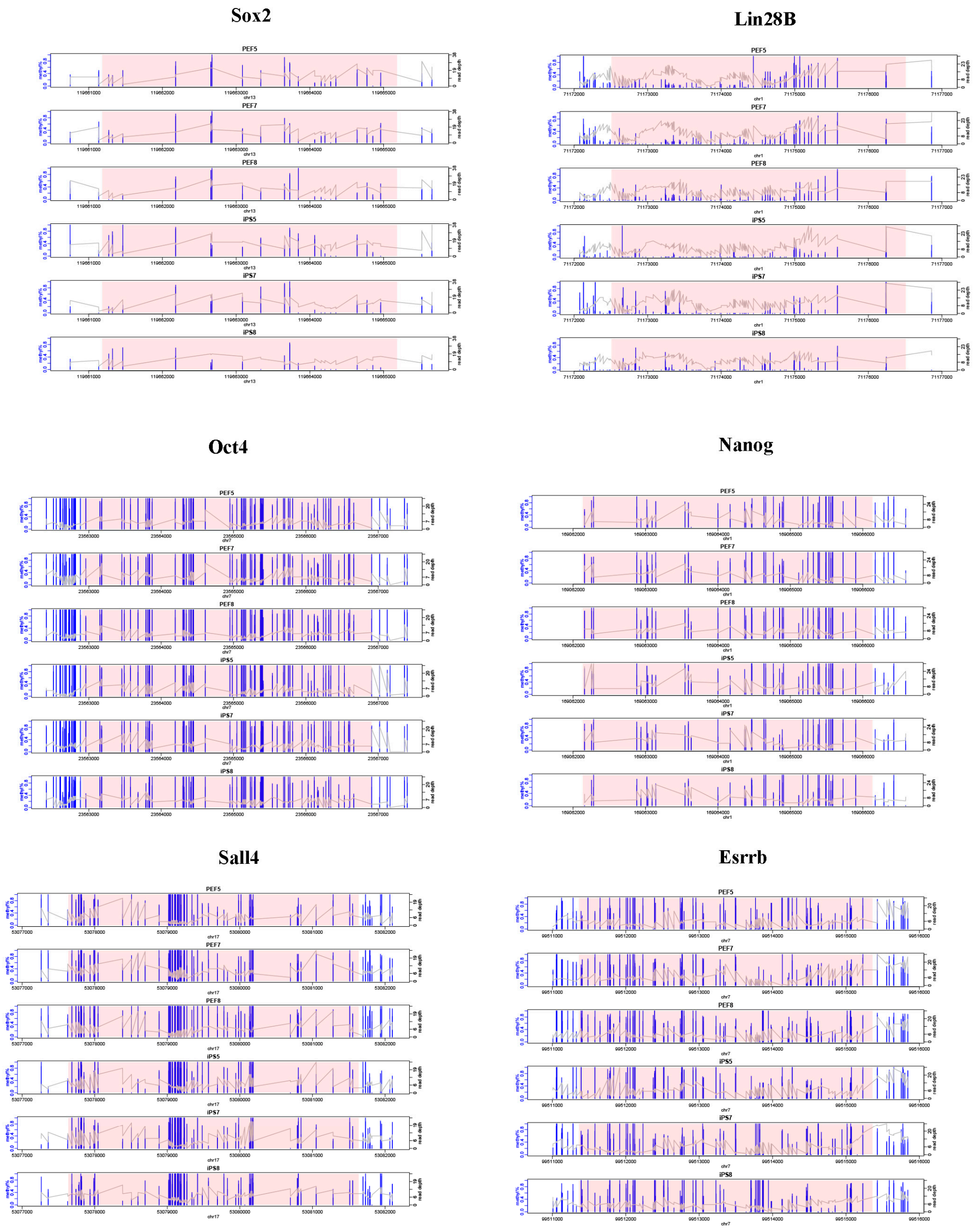

Figure S5 DNA methylation level of pluripotency genes

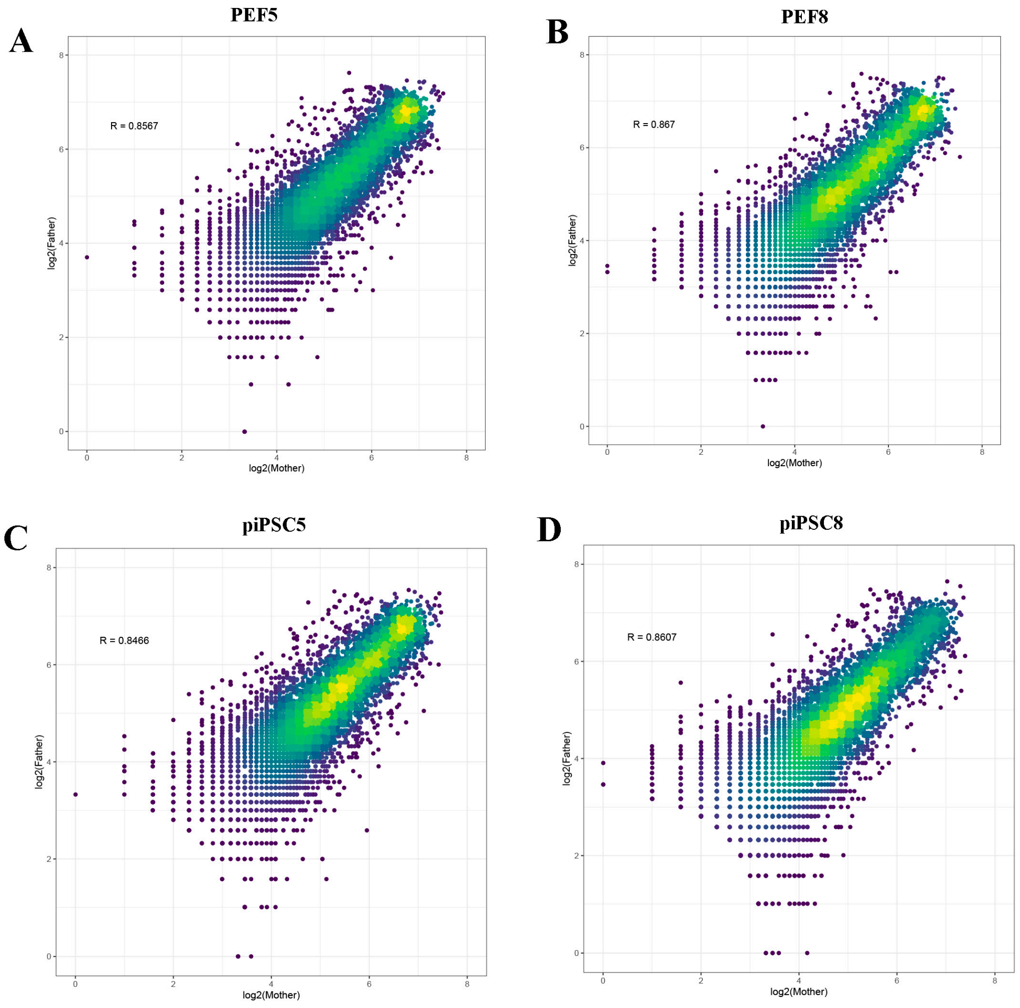

Figure S6 Scatter plots for RNA-seq reads assigned to each allele for SNP-containing genes. (**A**) Scatter plots for PEF7. (**B**) Scatter plots for PEF8. (**C**) Scatter plots for piPSC7. (**D**) Scatter plots for piPSC8.
