# Supplementary material for: The Defects of Epigenetic Reprogramming in Dox-Dependent Porcine-iPSCs
Source: Int J Mol Sci. 2022 Oct 8;23(19):11941. doi: 10.3390/ijms231911941 (PMC9570186; doi:10.3390/ijms231911941)
Supplement: Supplementary file 1 [file ijms-23-11941-s001.zip › Table S18.pdf]

Table S18 Sequence of RNA Oligonucleotides

Name	Sequences (5’-3’)
FOSL1 shRNA	GGGTGCCACACTAACCAACAT
RUNX1 shRNA	TACTCAGCTGAACTGAGAAAT
GLIS1 shRNA	AGGAAGTTTCCACTCCATCCA
SMARCAD1 shRNA	AAGGGTGATAGAGTCGTATTA
PRDM5 shRNA	AAGGCACCTACTTATACATAA
shRNA negative control	GTTCTCCGAACGTGTCACGT
