# Supplementary material for: The Defects of Epigenetic Reprogramming in Dox-Dependent Porcine-iPSCs
Source: Int J Mol Sci. 2022 Oct 8;23(19):11941. doi: 10.3390/ijms231911941 (PMC9570186; doi:10.3390/ijms231911941)
Supplement: Supplementary file 1 [file ijms-23-11941-s001.zip › Table S19.pdf]

Table S19 Primers sequences for qRT-PCR

Gene name		Primer sequence (5'-3')
FOSL1	F:	AGCAGAAGTTCCACCTCGTG
	R:	CTCGGGGCTGATCTGTTCAC
RUNX1	F:	GGTCGGAGTGGAAGAGGGAA
	R:	TCGGAAAAGGACAAGCTCCC
GAPDH	F:	AGGACCAGGTTGTGTCCTGT
	R:	CTTACTCCTTGGAGGCCATGT
BGN	F:	CAAGCTGTACAGGCTGGGC
	R:	TTGAAGAGGCTGATGCCGTT
FBN1	F:	CCATGCCAAGTCGATCCCAT
	R:	CCAGGCGTATATCCAGGCAG
COL1A2	F:	CTGGTCTTGGCGGGAACTTT
	R:	AGGACCAGTCTGACCAGGTT
THY1	F:	GCTCTCTTGCTAACAGTCTTGC
	R:	TGGTGGTATTCTCATGGCGG
POU5F1	F:	CGGGCTAGAGAAGGATGTGG
	R:	GGCAGGCACCTCAGTTTGAA
SOX2	F:	CATGAAGGAGCACCCGGATT
	R:	ATCATGCTGTAGCTGCCGTT
ESRRB	F:	ATGCCTCAAAGTGGGGATGC
	R:	TTTTAGTCAATGGCTTCTTCGCA
SALL4	F:	CCCCAACACATCAACTCGGA
	R:	ACTCGGCACAGCATTTCTCA
LIN28B	F:	CAGCCCCTTAAGGATCCGAG
	R:	CTTTGCTTGCCCCGCCTTC
NANOG	F:	AGGGCTCAGCCAGTACAGAA
	R:	CCAGCTCTGATTACCCCACA
