# Supplementary material for: The Defects of Epigenetic Reprogramming in Dox-Dependent Porcine-iPSCs
Source: Int J Mol Sci. 2022 Oct 8;23(19):11941. doi: 10.3390/ijms231911941 (PMC9570186; doi:10.3390/ijms231911941)
Supplement: Supplementary file 1 [file ijms-23-11941-s001.zip › Table S2.docx]

**Table S2 Basic information of ATAC-seq**

|  | PEF5 | PEF7 | PEF8 | piPSC5 | piPSC7 | piPSC8 |
| --- | --- | --- | --- | --- | --- | --- |
| Raw reads | 98,995,400 | 96,274,222 | 102,836,942 | 77,274,792 | 81,004,184 | 85,423,432 |
| Clean Reads | 90,807,182 | 86,822,382 | 95,041,522 | 71,405,830 | 75,245,114 | 79,551,040 |
| Clean reads Rates (%) | 91.73 | 90.18 | 92.42 | 92.41 | 92.89 | 93.13 |
| Clean Q30 Rates (%) | 94.35 | 94.61 | 94.44 | 94.49 | 94.62 | 94.65 |
| Mapped Reads | 78,743,074 | 74,076,430 | 84,509,988 | 57,366,920 | 58,539,144 | 55,315,441 |
| Mapping Rates (%) | 86.71 | 85.32 | 88.92 | 80.34 | 77.8 | 69.53 |
| Unique Mapped Reads | 28,047,190 | 40,623,371 | 30,136,821 | 45,499,272 | 44,156,792 | 41,040,409 |
| Unique Mapped Ratio (%) | 30.89 | 46.79 | 31.71 | 63.72 | 58.68 | 51.59 |
| Insert Size (bp) | 161.54 | 174.59 | 180.85 | 176.96 | 179.58 | 180.26 |
